# Apoptosis and Autophagy in Picornavirus Infection

**DOI:** 10.3389/fmicb.2019.02032

**Published:** 2019-09-03

**Authors:** Di Sun, Xingjian Wen, Mingshu Wang, Sai Mao, Anchun Cheng, Xiaoyao Yang, Renyong Jia, Shun Chen, Qiao Yang, Ying Wu, Dekang Zhu, Mafeng Liu, Xinxin Zhao, Shaqiu Zhang, Yin Wang, Zhiwen Xu, Zhengli Chen, Ling Zhu, Qihui Luo, Yunya Liu, Yanling Yu, Ling Zhang, Xiaoyue Chen

**Affiliations:** ^1^Institute of Preventive Veterinary Medicine, Sichuan Agricultural University, Chengdu, China; ^2^Key Laboratory of Animal Disease and Human Health of Sichuan Province, Sichuan Agricultural University, Chengdu, China; ^3^Avian Disease Research Center, College of Veterinary Medicine, Sichuan Agricultural University, Chengdu, China

**Keywords:** picornavirus, apoptosis, autophagy, cellular signaling, viral replication

## Abstract

Cell death is a fundamental process in maintaining cellular homeostasis, which can be either accidental or programed. Programed cell death depends on the specific signaling pathways, resulting in either lytic or non-lytic morphology. It exists in two primary forms: apoptosis and autophagic cell death. Apoptosis is a non-lytic and selective cell death program, which is executed by caspases in response to non-self or external stimuli. In contrast, autophagy is crucial for maintaining cellular homeostasis via the degradation and recycling of cellular components. These two mechanisms also function in the defense against pathogen attack. However, picornaviruses have evolved to utilize diverse strategies and target critical components to regulate the apoptotic and autophagic processes for optimal replication and the release from the host cell. Although an increasing number of investigations have shown that the apoptosis and autophagy are altered in picornavirus infection, the mechanism by which viruses take advantage of these two processes remains unknown. In this review, we discuss the mechanisms of picornavirus executes cellular apoptosis and autophagy at the molecular level and the relationship between these interactions and viral pathogenesis.

## Introduction

Picornaviruses are divided into 47 genera and are known for causing a wide variety of diseases, including numerous human and animal pathogens (Zell et al., 2017). The genus *Enterovirus* (EV) of the *Picornaviridae* family contains poliovirus (PV), coxsackievirus (CV), rhinovirus (RV), and numerous enteroviruses (bovine enterovirus, porcine enterovirus, and simian enterovirus), and most of picornaviruses are well-studied viruses. These viruses cause a broad spectrum of diseases, ranging from hand-foot-and-mouth infections and diarrhea to paralysis and encephalitis. Except for enteroviruses, other picornaviruses are causative agents of diseases and have a substantial impact on health care and economy, for example, encephalomyocarditis virus (EMCV), foot-and-mouth disease virus (FMDV) and hepatitis A virus (HAV).

Picornaviruses are small non-enveloped RNA viruses containing a positive-stranded 7–8 kb RNA genome. The genome consists of an integral ORF, a 5′ UTR, and a 3′ UTR with a poly(A) tail. The ORF is translated into a polyprotein, which is proteolytically processed into individual viral proteins containing VP1, VP2, VP3, VP4, leader proteinase (in the genus *Aphthovirus*), 2A, 2B, 3A, 3B, 3C, and 3D. These mature and functional proteins participate in viral translation, transcription, replication, assembly, and release. Due to their limited proteins, picornaviruses rely on the host cellular factors for efficient replication, and release. Hence, some of the most pivotal host mechanisms that typically affected by virus infection are pathways involved in cellular responses against environmental stress and cell death. Known as two critical processes contributing to the maintenance of cellular homeostasis, autophagy controls the turnover of organelles and proteins within cells, and apoptosis is the principal mechanism by which unwanted or abnormal cells are physiologically eliminated from organisms. Picornaviruses can subvert autophagy and apoptosis by selectively exploiting some key cellular factors, which are indispensable pathways to cell fate and normal cellular functions. This article discusses the possible molecular mechanism by which picornaviruses modulate apoptosis and autophagy, and the potential role of these two pathways in viral replication and spread.

## The Life Cycle of Picornaviruses

The life cycle of picornaviruses starts when viruses bind to cell surface receptors, leading to receptor-mediated endocytosis. However, different picornaviruses utilize different mechanisms to induce the endocytic process. Although a member of the genus *Enterovirus*, infection of HRV14 requires clathrin-mediated endocytosis for infection, poliovirus does not depend on the clathrin-mediated pathway (DeTulleo and Kirchhausen, 1998). Virus uncoating releases the viral genome via a pore in the endosomal membrane into the cytoplasm. Afterward, viral RNA is delivered to the cytosol, it is translated into a large polyprotein and subsequently processed by viral proteinases into single functional proteins including capsid proteins (VP0, VP1, and VP3) and non-structural proteins (2A–2C and 3A–3D). Viral genome replication relies on the membranous replication organelles (ROs) (van der Schaar et al., 2016). During infection, EMCV manipulates PI4KA, while enteroviruses depend on PI4KB for the formation of ROs (Dorobantu et al., 2015). Viral RNA-dependent RNA polymerase (3D^pol^) is required for genome replication, which synthesizes a negative-stranded RNA that serves as a template for the synthesis of new positive-stranded RNA. After that, newly synthesized viral RNA serves as a template for translation and replication. Viral capsid proteins assemble into viral particles and then form virions with nascent viral RNA. To favor viral replication and propagation, picornaviruses selectively alter cellular pathways, such as, by inducing the shutoff of host protein translation, inhibiting the immune response and modulating cell death processes. These processes are mostly achieved by viral non-structural proteins, especially proteinase, including 2A protease and 3C protease. Picornaviruses are typically considered as cytolytic viruses as host cells are lysed for virus release. Nonetheless, an increasing of investigations has demonstrated that non-enveloped picornaviruses can also adopt a non-lytic transmission strategy via vesicles, which require autophagy to form double-membrane autophagosomes carrying virions to the extracellular environment (Feng et al., 2013).

## Picornavirus and Apoptotic Pathway

### Introduction to Apoptosis

Apoptosis is a non-inflammatory type of programed cell death (PCD) and requires a cascade of signaling proteins to respond to the activation of a death signal. This process is characterized by facilitating morphological changes including cell shrinkage, chromatin condensation, and plasma membrane blebbing (Galluzzi et al., 2012). The intrinsic mitochondrial pathways and the extrinsic pathway (also called death receptor) are two distinct but ultimately converging pathways for apoptosis. Caspases (a family of cysteine aspartyl-specific protease) are involved in the intrinsic and the extrinsic pathways, and the activation of caspase 3 is an executioner to induce apoptotic cell death. Host cells utilize apoptosis as a defense strategy to respond to invading pathogens. Simultaneously, viruses have evolved to subvert cell death signaling pathways. For example, PV was reported to suppress apoptosis for efficient virus production in infected cells (Tolskaya et al., 1995).

### Apoptosis Machinery

In broad terms, there are two distinct signaling cascades trigger the apoptotic pathway, the extrinsic cell apoptotic pathway and the intrinsic apoptotic pathway. The initiation of the extrinsic pathway is induced by the recognition of cognate ligands, such as the FAS-ligand receptor and tumor necrosis factor receptor (TNFR). Subsequently, the death receptors recruit adaptor proteins, including TNFR-associated death domain (TRADD) and FAS-associated death domain (FADD) to form complexes in the mitochondria or cytoplasm. After recruitment, these complexes activate intracellular caspase 8, leading to activation and translocation of caspase 3 and execution of apoptotic cell death. In contrast, intracellular stresses (DNA mutation or ER stress) activate the intrinsic pathway by inducing a loss of mitochondrial outer membrane permeability (MOMP). This process leads to the efflux of pro-apoptotic proteins into the cytoplasm, such as cytochrome *c*, apoptosis-inducing factor (AIF), and Smac/DIABLO (Er et al., 2006). Many studies have revealed that MOMP leads to apoptosis dependent on caspase activity; however, MOMP could contribute to cell death in a caspase-independent manner (Tait and Green, 2010). The permeabilization of the outer mitochondrial membrane relies on the activation, translocation, and oligomerization of the multidomain B cell lymphoma 2 (Bcl-2) family proteins. As essential regulators, this family proteins can be divided into two classes: pro-apoptotic (Bax, Bak, Bad, and Bid) and anti-apoptotic (Bcl-2 and Bcl-xL), which dynamically regulate apoptosis. When caspase 8 cleaves the Bcl-2 protein Bid, truncated Bid (tBid) is formed and can activate two Bcl-2 proteins (BAX/BAK), resulting in their translocation to the mitochondria to induce the intrinsic apoptotic pathway (Yoshida et al., 1998; Zhang et al., 2000). BAX/BAK is required for caspase activation, regarded as a critical gateway in the intrinsic apoptotic pathway (Ruiz-Vela et al., 2005). The Bcl-2 proteins oligomerize on the mitochondrial membrane forming a pore and inducing the release of cytochrome *c* into the cytoplasm. The binding of cytochrome *c* and APAF-1 recruits and catalyzes procaspase 9 into caspase 9, leading to the activation of caspase 3 and apoptosis (Slee et al., 1999).

### The Role of Apoptosis in Picornavirus Replication

The host cells have distinct mechanisms to detect the presence of pathogens and then defense against them. To survive within cells, picornaviruses have evolved diverse strategies and targeted critical components in the apoptotic cascade, to disrupt the induction of apoptosis process. For example, picornaviral proteases operate the cleavage of caspases and pro-apoptotic proteins and subvert nuclear-cytoplamic trafficking to suppress the host apoptosis. Several picornaviruses, such as PV, CVB3, FMDV, and EMCV, have been reported to trigger host apoptosis but also subvert execution of apoptosis to promote viral replication. Each of these viruses has shared specific targets or distinct mechanisms to modulate the host apoptotic response. The initiation of the antiviral response induces an antiviral transcriptional program that is composed of the expression of cytokines, chemokines, interferons (IFNs) and the activation of cell death pathways (apoptosis and necroptosis). IFN pathways, which are composed of viral sensors (RIG-I, MDA-5, TLR-3), adaptor proteins (MAVS, TRIF, and STING), kinases, and transcription factors (IRF3 and IRF7), antagonize viral infection by facilitating the expression of ISGs (Schoggins, 2014). In picornavirus infection, endosomal Toll-like receptor (TLR-3), a PRRs, recognizes and binds to double-stranded viral RNA (dsRNA), inducing the exposure of the Toll-IL-1 receptor (TIR) domain (Alexopoulou et al., 2001; de Bouteiller et al., 2005). TIR domain-containing adaptor inducing beta interferon (TRIF) binds to TLR-3 activating IRF-3, TANK binding kinase-1 (TBK-1) and NF-κB, which are transcription factors essential for IFN- β production. The second set of PRRs includes RIG-I and melanoma differentiation-associated gene-5 (MDA5), which recruit adaptor MAVS (also known as IPS-1 and VISA) via their intracellular RNA helicase activity and subsequently activate TBK-1, IRF-3 and NF-κB, leading to the phosphorylation and dimerization of IRF-3 (Kawai et al., 2005; Slater et al., 2010). Then, IRF-3 homodimers translocate to the mitochondrial membrane and complex with Bax, resulting in the formation of a pore and efflux of cytochrome *c*. TRIF was found to induce cell death and the initiation of this process relies on the FADD-caspase 8 axis (Kaiser and Offermann, 2005). Previous studies have shown that the TRIF/interacting protein (RIP)-1/caspase-8 complex is necessary for dsRNA-mediated TLR-3-dependent apoptosis, the interaction of TRIF with RIPK1 through its C-terminal RIP homotypic interaction motif (RHIM), and the trigger of apoptosis was in a caspase-8 dependent manner (Kaiser and Offermann, 2005; Estornes et al., 2012). Furthermore, the activation of IRF-3 by RIG-I is regulated by the caspase8-mediated cleavage of the RIPK1 protein (Rajput et al., 2011).

#### Picornaviral 2B Protein and Apoptosis

**FIGURE 1:**
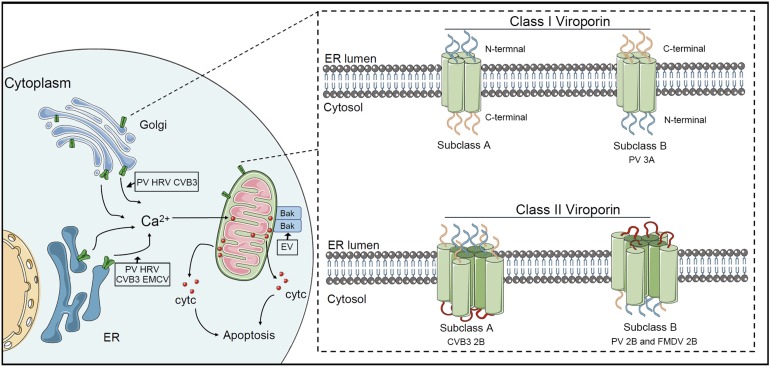
Picornaviral 2B protein regulates apoptosis. The 2B protein of PV, HRV, and CVB3 directly targets Golgi and ER membrane and assembles pores in the Golgi membrane and ER membrane, which induces the release of Ca^2+^ from organelles into the cytosol. The mitochondrion takes up Ca^2+^, leading to the release of cytochrome *c* and the formation of apoptosome. Different from these three picornaviruses, EMCV 2B protein only alters the Ca^2+^ content in the ER. EV71 2B protein targets the pro-apoptotic protein Bax to trigger the activation of the mitochondrial cell death pathway. There are two major groups viroporin, class I (containing one transmembrane domain) and class II (containing two transmembrane domains). CVB3 2B protein belongs to class IIA, while PV 2B and FMDV 2B belong to class IIB.

The picornaviral 2B protein is a small hydrophobic membrane protein, which exhibits viroporin or viroporin-like activity. Viroporins are divided into two major groups, class I (containing one transmembrane domain) and class II (containing two transmembrane domains). The viroporins of class IIA have lumenal N and C termini, whereas viroporins of class IIB have cytosolic N and C termini (Figure 1) (Nieva et al., 2012). The 2B protein of CVB3 belongs to class IIA and consists of a hydrophobic helix and a cationic amphiphilic helix (van Kuppeveld et al., 1996; de Jong et al., 2004). However, 2B proteins of PV and FMDV belong to class IIB (Agirre et al., 2002; Ao et al., 2015; Gladue et al., 2018). HAV 2B protein has a viroporin-like region that interacts with cellular membranes (Shukla et al., 2015). Picornaviral 2B protein involves in increasing membrane permeability, altering Ca^2+^ homeostasis and modulating host apoptosis and autophagy.

ER stress induces calcium (Ca^2+^) release from ER stores and Ca^2+^ accumulation in the mitochondrial matrix, which requires the 1,4,5-triphosphate receptor (IP3R) and voltage-dependent anion channel (VADC) as mediators. This increases in the mitochondrial Ca^2+^ concentration contributes to the increase in MMP; therefore, the movement of mitochondrial pro-apoptotic proteins is promoted, which triggers cell death (Deniaud et al., 2008). An early study showed that a notable increase in intracellular Ca^2+^ concentration was observed in PV infection (Irurzun et al., 1995). Fluorescent Ca^2+^ indicators were used to determine that the CVB3 2B protein manipulated intracellular Ca^2+^ homeostasis, including by decreasing Ca^2+^ content in the ER and Golgi, which failed to suppress apoptosis (Campanella et al., 2004). Notably, subsequent studies revealed that the expression of 2B protein in PV and HRV resulted in a significant decrease in Ca^2+^ in the ER and Golgi complex; however, EMCV 2B protein decreased only in the ER [Ca^2+^] while HAV 2B protein did not alter the Ca^2+^ content in the ER and Golgi (de Jong et al., 2008) (Figure 1). The release and translocation of Ca^2+^ from the ER have been demonstrated to be involved in PV-induced apoptosis (Brisac et al., 2010). Increased cytosolic Ca^2+^ not only leads to the activation of calcium-dependent cell death pathways, but also induces autophagy. In BHK-21 cells, FMDV 2B protein disrupted the Ca^2+^ concentration, which was implicated in the induction of autophagy (Ao et al., 2015). Recent studies have revealed the potential functions of picornaviral 2B, identified as a viroporin, which oligomerizes to form aqueous pores on the host cellular membrane (Fung et al., 2015). A previous study demonstrated that EV71 infection induces apoptosis by the mitochondrial pathway, in which the release of cytochrome *c* from the mitochondria and cleavage of caspase 9 can be observed in multiple cell lines (Chang et al., 2004). A subsequent study revealed that EV71 2B protein could induce conformational changes in the pro-apoptotic protein Bax to trigger the activation of the mitochondrial cell death pathway (Cong et al., 2016).

### Picornaviral 3C Protease and Apoptosis

**FIGURE 2:**
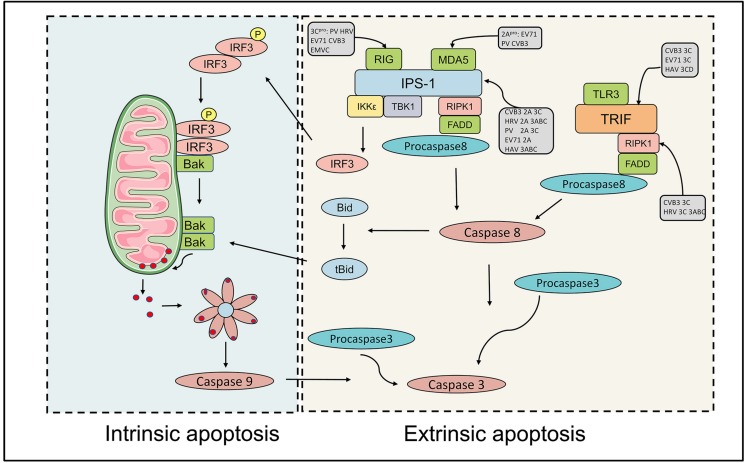
Effects of picornavirus infections on those intrinsic and extrinsic apoptotic pathways. Caspase 9-dependent intrinsic apoptosis is initiated by the release of mitochondrial cytochrome *c* into the cytoplasm. When activated, the RNA sensors RIG-I and MDA-5 associate with IPS-1 and recruit TBK1 and IKKγ a death receptor complex in mitochondria. TBK1 and IKKγ phosphorylate IRF-3 dimers, which then induce a pore in the mitochondrial membrane, allowing the release of cytochrome *c* into the cytoplasm, which induces intrinsic apoptosis. RIG-I is cleaved by 3C protease in cells infected with PV, HRV, EV1, EV71, EMCV and CVB3, while MDA-5 is a target of enteroviral 2A protease. The adaptor MAVS is degraded by viral proteases of PV, EV71, HRV, CVB3, and HAV. The extrinsic apoptosis requires the formation of the caspase 8/RIPK1/FADD/IPS-1 complex. RIPK1 is also a target for enteroviral 3C protease, which disrupts extrinsic apoptosis.

Picornaviral 3C protease, a chymotrypsin-like cysteine protease, exists in all picornaviruses. Despite cooperating with leader protease and 2A protease in some genera, 3C protease and its precursor are responsible for the most primary cleavages and secondary cleavages of polyproteins. 3C protease is capable of regulating the apoptotic process via protease activity toward multiple cellular factors. To attenuate the innate response, the RNA sensor RIG-I is cleaved by 3C protease in cells infected with PV, HRV, EV1, EV71, EMCV and CVB3, while adaptor MAVS is degraded in PV, EV71, HRV, CVB3, and HAV (Table 1). Notably, MAVS proteolysis in HAV requires both mitochondrial targeting property of 3A and the cysteine protease activity of 3C protease (Yang et al., 2007). In contrast to RIG-I, MDA-5 can be cleaved in PV-infected cells in proteasome and caspase-dependent manner instead of by viral proteases (Barral et al., 2007). Subsequent studies reported that MDA-5 was a target of enteroviral 2A protease (Feng Q. et al., 2014). EV 71 3C protease cleaves IRF7 to compromise the host innate immune response (Lei et al., 2013). The formation of the caspase 8/RIPK1/FADD/IPS-1 complex is essential for the induction of extrinsic apoptosis. Previous researches reported that RIPK1 could be cleaved by caspase 8 to strengthen the interaction of this complex in a pro-apoptotic manner (Lin et al., 1999). Interestingly, RIPK1 is also a target for HRV 3C protease, which might inhibit extrinsic apoptosis (Croft et al., 2018; Lötzerich et al., 2018) (Figure 2). EMCV 3C protease can cleave Ras-Gap SH3 domain-binding protein 1 (G3BP1) disrupting the formation of SGs, which are involved in regulating IFN-β gene expression (Ng et al., 2013). Similar disruption was described in PV- and CV-infected cells (White et al., 2007; Langereis et al., 2013). This observation was also reported in FMDV-infected cells, which G3BP1 could be cleaved by 3C protease of FMDV (Galan et al., 2017). Moreover, subsequent research revealed that G3BP1 is a target of leader protease of FMDV and ERAV as well (Visser et al., 2019). Accumulating evidence suggests that different picornaviruses adopt multiple mechanisms to disrupt the immune response.

**TABLE 1:** The host proteins associated with apoptosis are cleaved by picornaviral proteases.

**Host protein**	**Function in apoptosis**	**Virus**	**Viral proteases**	**References**
RIG-I	A membrane-bound Toll-like receptor, dsRNA and ssRNA sensor.	PV HRV (1A,16) EMCV EV1 EV71 CVB3	3C 3C 3C 3C 3C 3C	Barral et al., 2009; Feng Q. et al., 2014 Barral et al., 2009 Barral et al., 2009; Papon et al., 2009 Barral et al., 2009 Lei et al., 2010; Feng Q. et al., 2014 Feng Q. et al., 2014
MDA5	A cytosolic innate immune receptor, dsRNA sensor.	PV EV71 CVB3	2A 2A 2A	Feng Q. et al., 2014 Feng Q. et al., 2014 Feng Q. et al., 2014
MAVS (also known as IPS-1, VISA and Cardiff)	A central adaptor protein in the innate immune response to RNA viruses.	PV HRV CVB3	3C, 2A 2A, 3ABC 2A, 3C	Drahos and Racaniello, 2009; Feng Q. et al., 2014 Drahos and Racaniello, 2009 Drahos and Racaniello, 2009; Mukherjee et al., 2011; Feng Q. et al., 2014
		HAV EV71	3ABC 2A	Yang et al., 2007; Drahos and Racaniello, 2009 Wang et al., 2013
TRIF	Toll/IL-1R (TIR)-domain-containing adaptor protein inducing IFN-β, which is essential for induction of TLR3. The RHIM domain mediates cellular apoptosis.	EV68 EV71 CVB3 HAV	3C 3C 3C 3CD	Xiang et al., 2014 Lei et al., 2011 Mukherjee et al., 2011 Qu et al., 2011
RIPK1	RIPK1 binds to adaptor proteins including TRIF, FADD, RIPK3, NEMO, and SQSTM1, which are involved in sensing RNA, NF-κB signaling, autophagosome formation, and apoptosis.	HRV CVB3	3C, 3ABC 3C 3C	Lötzerich et al., 2018 Croft et al., 2018 Wang et al., 2019
NEMO	A scaffolding component of the IκB kinase complex required for NF-κB activation	FMDV HAV	3C 3C	Wang et al., 2012 Wang et al., 2014
Sam68	A nuclear RNA-binding protein, involved in RNA metabolism, apoptosis, and signal transduction.	FMDV	3C	Lawrence et al., 2012
DAxx	A FAS ligand, involved in apoptosis, transcriptional control, innate immune antiviral response.	FMDV	L	Pineiro et al., 2012
PinX1	A telomere-binding protein, involved in cellular apoptosis.	EV71	3C	Li et al., 2017
SFPQ	A nuclear protein, involved in DNA repair, transcriptional regulation, splicing, RNA transport and apoptosis.	HRV	3C, 3CD	Flather et al., 2018

As an IRES *trans*-acting factor, polypyrimidine tract-binding protein (PTB) is present in a complex containing RNA-binding proteins NONO/p54nrb, hnRNPA1, hnRNPC1/C2, hnRNPA2/B1 and PSF/SFPQ, which is central in apoptotic cells. The alteration in a single protein with overexpression or siRNA knockdown is sufficient to modulate apoptosis rates, while the depletion of SFPQ or PTB resulted in reduced apoptosis (King et al., 2014). Interestingly, PTB has been reported to be cleaved by viral protease in PV, HRV, and HAV (Back et al., 2002; Kanda et al., 2010). Similarly, the proteolytic processing of PTB was observed in FMDV-infected cells (Rodriguez Pulido et al., 2007). Recently, it was found that SFPQ is characterized as a pro-viral factor, which is specifically targeted by HRV 3C protease for proteolysis (Flather et al., 2018). The cleavage of the far-upstream element (FUSE) binding protein 2 (FBP2) is observed in EV71-infected cells, and this cleavage is executed by 3C protease (Chen et al., 2013). EV 71 3C protease cleaves PinX1 at the Q51-G52 pair, a telomere-binding protein, disrupting telomere maintenance, which has been suggested to induce apoptosis for promoting virus release (Li et al., 2017).

The src-associated substrate in mitosis of 68 kDa (Sam68) has been characterized in multiple cellular processes associated with RNA usage and gene expression, including transcription, translation, RNA splicing and export (Li et al., 2002; Matter et al., 2002; Coyle et al., 2003). It was revealed that Sam68 could induce apoptosis via its RNA-binding ability. The specific-RNA binding-defective protein Sam68 G178E (Gly178 was substituted by Glu) could not promote apoptosis, which indicated that the induction of apoptosis required a fully-functional RNA-binding domain (Taylor et al., 2004). An alteration in the nuclear localization of Sam68 to the cytoplasm, which was related to the disruption of the NPC, was observed in the infection of PV, HRV2, HRV16, EV71, and FMDV (McBride et al., 1996; Lawrence et al., 2012; Zhang et al., 2014; Walker et al., 2015). However, the relocalization of Sam68 occurs before the complete disruption of the NPC during HRV infection, and redistribution of Sam68 is involved in PI3K/Akt activation in EV71 infection. In contrast to enteroviruses, FMDV 3C protease cleaves Sam68, resulting in the diffusion of the C-terminal portion into the cytoplasm and the attachment of viral IRES for translation (Lawrence et al., 2012). Remarkably, one of these predicted cleavage sites is Q179/G, which is close to the mutation site within RNA-binding-defective Sam68. It seems that different viruses utilize multiple strategies to disrupt the function of Sam68 including RNA binding or pro-apoptotic abilities for promoting viral infection, but the mechanisms remain unknown.

#### Other Viral Proteases and Apoptosis

In addition to 3C protease, leader protease in *Aphthovirus* and 2A protease in *Enterovirus* are pivotal non-structural proteins in the viral life cycle. FMDV leader protease is a papain-like cysteine protease, which exists in two forms (Laboratory and Lb) (Kirchweger et al., 1994). Different from FMDV leader protease, leader protein encoded in the *Cardiovirus* genus lacks the protease activity. According to structures and functions, picornaviral 2A proteins can be divided into five types (A) chymotrypsin-related protease, (B) *Aphthovirus*-like 2A, (C) hepatitis-A-virus-like 2A, (D) *Parechovirus*-like 2A, and (E) the 2A sequence of the genus *Cardiovirus* (Yang et al., 2017). The chymotrypsin-like 2A encoded in *Enterovirus* genus, service as a proteolytic enzyme processing viral polyprotein and host proteins, whose function is similar to FMDV leader protease. Here, we mainly discussed the potential mechanisms of viral proteases counteract the host immune response.

In some picornavirus infections, the induction and suppression of apoptosis regulate cell lysis and viral spread. It has been revealed that PV infection induces apoptosis early in infection and inhibits apoptosis during late-stage infection. In late-stage infection, PV 2A protease can suppress apoptosis by inducing the aberrant processing of procaspase-9 (Belov et al., 2003; Burgon et al., 2009). In contrast, CVB3 2A protease could induce caspase 8-mediated activation of caspase 3 and the release of cytochrome *c*, activating the caspase 9-mediated the intrinsic mitochondria-mediated apoptotic pathway (Chau et al., 2007). CVB3 2A protease is responsible for cleavage of death-associated protein 5 (DAP5), a structural homolog of eIF4G, resulting in the promotion of viral replication and host apoptosis (Hanson et al., 2016). EVA71 modulates the mitochondrial apoptotic pathway via the conformational activation of Bax (Han and Cong, 2017). Enteroviruses can induce apoptosis, and disruption of the NPC was observed during infection. However, there is little evidence that explains the link between alterations in NPC and apoptotic cell death. The PV 2A protease was responsible for degrading Nup 62, Nup 98 and Nup 153, while HRV 2A protease cleaved endogenous Nup 98 and recombinant Nup 62 (Park et al., 2008; Castello et al., 2009). The cleavage of Nups leads to diverse consequences in trafficking pathways, including the lack of nuclear import receptor cargo complexes and nuclear envelope leakiness. Transient expression of the 2A protease of EV 71 is sufficient to trigger apoptosis (Kuo et al., 2002).

The FMDV leader protease inhibits host innate immune response by multiple mechanisms. Death-domain associated protein (Daxx), a Fas ligand, involves multiple cellular mechanisms, including apoptosis, transcriptional control, and innate immune response (Yang et al., 1997). In FMDV-infected cells, Daxx is reported to be proteolysed by leader protease, which evades the host anti-viral response (Pineiro et al., 2012). Picornaviral proteases target the host innate immune sensors subverting the specific recognition of viral RNA. Similar to enteroviral 3C protease and 2A protease cleaving RIG-I and MDA-5 respectively, FMDV leader protease can cleave laboratory of genetics and physiology 2 (LGP2), which is regarded as another RIG-I-like receptor in recognizing viral RNA in antiviral immunity (Rodriguez Pulido et al., 2018).

## Autophagy in Virus Infection

### Overview of Autophagy

**FIGURE 3:**
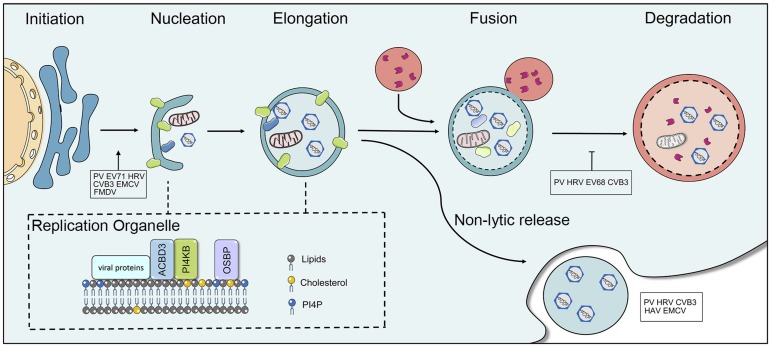
The interaction between picornaviruses and the host autophagy pathway. Autophagy is a five-step preprocess, including induction, nucleation, expansion (elongation), fusion, and cargo degradation/metabolic recycling. Picornaviruses have been shown to subvert the host autophagy machinery for viral replication and non-lytic release. Some viruses, including PV, CVB3, EV71, HRV, FMDV, and EMCV have been reported to utilize the autophagy for optimal infection. Picornaviruses replicate on membranous replication organelles, which require a complex containing viral (2B and 3A), ACBD3, OSBP, and PI4KB (or PI4KA). The fusion of autophagosome with lysosomes is inhibited by picornaviruses. HAV, CVB3, PV, HRV, and EMCV can trigger autophagy and release virions in single-membrane vesicles.

The ubiquitin-proteasome system (UPS) and the autophagy-lysosome system are the two major pathways that participate in cellular protein quality control in eukaryotic cells and are crucial for maintaining cellular homeostasis via the degradation and recycling of cellular components (Lilienbaum, 2013). Dysfunctions in autophagy cause a wide range of illnesses, including neurodegenerative disorders, cancer, cardiovascular, and inflammatory bowel disease. The degradation of autophagy is considered to be non-apoptotic cell death. Three types of autophagy have been identified in mammalian cells (macroautophagy, microautophagy, and chaperone-mediated autophagy). Macroautophagy (hereafter referred to as autophagy) is an evolutionarily conserved process, in which the cytoplasmic components are sequestered within cytosolic double-membrane vesicles and then delivered to lysosomes for degradation (Mizushima and Klionsky, 2007; Klionsky et al., 2011). Autophagy is a five-step preprocess, induction, nucleation, elongation (expansion), fusion, and cargo degradation/metabolic recycling. More than 30 autophagy-related genes (Atg) participate in different stages of the execution of autophagy (Mizushima et al., 2002; Klionsky, 2007). Autophagy is initiated by the formation of crescent-shaped isolated-membrane vesicles sequestering dysfunctional organelles, misfolded protein and invasive microbes, which are known as isolation membranes or phagophores. Subsequently, the phagophore grows and the phagophore edge fuses to form an enclosed double-membrane vesicle called the autophagosome. Following elongation, the outer membrane of the autophagosome finally fuses with lysosomes to form autolysosomes, while lysosomal enzymes degrade the contents and inner membrane of the autophagosome (Figure 3). The sequestration of cargo within cytosolic double-membrane vesicles has been regarded as the most identifiable feature of macroautophagy.

### Autophagic Machinery

Autophagic, proteasome and cytosolic degradation achieve the maintenance of cellular activity and viability via balancing biosynthesis and turnover. Autophagy responds to various conditions of stress through regulatory factors composed of different sets of Atg proteins (Feng Y. et al., 2014). The first autophagy-specific gene, Atg1, was identified as a Ser/Thr protein kinase and is required for initiation (Matsuura et al., 1997). The ULK1 (uncoordinated 51-like autophagy activating kinase) and FIP200 are two mammalian homologs of Atg1 (Jung et al., 2009). The process of autophagy is achieved by several functional complexes including the Atg/Ulk1 complex, the PtdIn3K complex, and two ubiquitin-like conjugation systems (Wong et al., 2013), the Atg12-Atg5 conjugation system and microtubule-associated protein light chain 3 (LC3-Atg8) conjugation system (Ravikumar et al., 2010). Two essential protein complexes involved the formation of autophagosomes are Ulk1 and PI3KC3-C1. In response to intracellular or extracellular stress and signals, including starvation, ER stress, and pathogen infection, autophagy can be induced. In yeast and *Drosophila*, the target of rapamycin (TOR) can negatively regulate Atg1 (Chang and Neufeld, 2009). Rapamycin treatment and starvation could induce TOR inhibition in yeast. Then, the kinase activity of Atg1 is stimulated and the binding affinity to Atg13 and Atg 17 is increased, which facilitates the initiation of autophagosomes by increasing the scaffold formation of Atg1-Atg13-Atg17 complex and the recruitment of Atg proteins (Kamada et al., 2000; Kabeya et al., 2005). The inhibition of the mammalian target of rapamycin (mTOR) is inhibited when starvation induces autophagy, which is an essential regulator integrating nutrient signals and growth factors (Kuma et al., 2004). The mTORC1 negatively regulates a complex formed of ULK1, Atg13, Atg101, and FIP200 in response to growth factors that trigger the PI3K-AKT pathway or other nutrient-related signals.

The PtdIns3K complex, comprising Vps34, Vps15, Vps30, and Atg14, is involved in the vesicle nucleation and the recruitment of PtdIns3P-binding proteins. Beclin 1, the mammalian homolog of yeast Vps30/Atg6, forms a complex with PtdIn3K and localizes on the *trans*-Golgi network (TGN) membrane that is involved in autophagic vesicle nucleation (Kihara et al., 2001). The connection between the molecular mechanism of autophagy and human disease was first identified regarding the autophagy-promoting function of Beclin 1 in inhibition of tumorigenesis (Liang et al., 1999). Furthermore, a series of studies revealed that protein expression of Beclin 1 is linked to cell growth and death, neurodegeneration, including Alzheimer’s disease and Huntington’s disease, and pathogen infection (Liang et al., 1998; Yu et al., 2004; Boya et al., 2005; Rubinsztein et al., 2005; Orvedahl et al., 2007; Levine and Kroemer, 2008).

For Ub1 protein conjugation, there are eight homologs of Atg8 at least in mammalian cells. LC3 and GATE-16/GABARAP are two subfamilies for these homologs. They are both essential in autophagosome biogenesis; however, LC3s participate in the elongation of the phagophore membrane whereas the GABARAP subfamily is involved in later steps in autophagosome maturation (Weidberg et al., 2010). The C-terminal fragment of proLC3 is proteolytically cleaved by Atg4 to generate cytosolic LC3-I with an exposed lipid conjugation site, followed by its subsequent activation and transformation by Atg7 and Atg3, respectively. Consequently, LC3 conjugates with the cellular lipid phosphatidylethanolamine (PE), forming LC3-II (PE-conjugated form) (Kabeya et al., 2004). The formation of discrete puncta, which are indicative of the conversion of LC3-I to LC3-II and can be observed via immunofluorescence analysis, is considered a critical regulatory step of autophagosome formation. The control of the initiation of autophagosome formation by modulating the mTOR pathway and LC3 lipid conjugation and the control of processing from the maturation of autophagosomes into degradative autolysosomes are two main strategies to regulate autophagy.

Initially, autophagy was described as a random process balancing cellular components in response to different types of stress. However, there is accumulating evidence that autophagy can regulate and degrade protein aggregates and organelles in a selective manner (Glick et al., 2010; Gatica et al., 2018). Instead of taking up and degrading random cytoplasm into phagophores for non-selective autophagy, the membrane-bound vesicle removes specific components via selective autophagy (Jin et al., 2013). The well-characterized molecule, sequestosome 1 (SQSTM1)/p62 molecule is a crutial autophagy receptor protein in bridging ubiquitylated substrates to the nascent autophagic vesicles in mediating selective autophagy (Johansen and Lamark, 2011; Stolz et al., 2014). SQSTM1 mutations were identified as being linked to Paget’s diseases, in which increased and disorganized bone turnover lead to focal abnormalities (Ralston and Layfield, 2012).

### Role of Autophagy in Viral Replication

Autophagy is a self-degradative process to maintain cellular homeostasis. In *Picornavirus* family, many viruses have been reported to utilize the autophagic machinery for optimal infection, including PV, CVB3, EV71, HRV, FMDV, and EMCV (Wong et al., 2008; Huang et al., 2009; Kemball et al., 2010; Klein and Jackson, 2011; O’Donnell et al., 2011; Zhang et al., 2011). The increased autophagosome formation was observed at the early stage of CVB3 infection, while the decreased autophagosome formation along with increased apoptosis was observed at the late stage of infection. It was presumed that the replication of CVB3 depended on the autophagosome, and the viral release relied on apoptosis (Xin et al., 2014). Studies on enteroviruses showed that autophagy was inhibited at the stage of autolysosomal degradation (Jackson et al., 2005). During CVB3 infection, increased protein polyubiquitination and the accumulation of protein-ubiquitin conjugates were observed in cells and tissues, revealing that CVB3 might take advantage of the UPS for pathogenesis (Luo et al., 2010). Moreover, it was suggested that CVB3 infection could block autolysosomal degradation due to the accumulation of autophagosomes, which causes the protein levels of LC3-II and p62 to increase (Wong et al., 2008; Kemball et al., 2010). Subsequent studies have revealed that CVB3 infection leads to the cleavage of SQSTM1 at amino acid 241 within the tumor necrosis-associated factor 6 binding (TB) domain and viral 2A protease is responsible for this cleavage (Shi et al., 2013). After that, SQSTM1 was later found to regulate viral replication, and the knockdown of SQSTM1 caused an increase in the production of viral protein and viral titers. Increasing studies have shown that SQSTM1 can interact with the CVB3 capsid protein VP1. The cleavage of SQSTM1 has been observed in multiple enteroviruses, including infection of PV, HRV, and EV D68, and it has been suggested that this is a common strategy to interact with cargo adaptors (Corona et al., 2018). The soluble *N*-ethylmaleimide-sensitive factor attachment protein receptor (SNARE) proteins are required for the fusion of the autophagosome with the endosome/lysosome (Itakura et al., 2012). One of these SNARE proteins, syntaxin 17 (STX17), could interact with the synaptosomal-associated protein 29 (SNAP29), which mediates fusion between the outer autophagosomal membrane and lysosomal membrane (Itakura and Mizushima, 2013). CVB3 infection was shown to decrease the transcription and translation of STX17, impairing the autophagic flux. It was also found that the overexpression of STX17 could restore lysosomal function and blocked apoptosis induced by CVB3 infection (Tian et al., 2018). Lang proposed that the blockage of autophagosome-lysosome fusion is the reason for the accumulation of autophagosomes in CVB3-infected cells and that SNARE complexes are potential targets. Subsequently, it was determined that CVB3 infection inhibits autophagic flux via impairing the formation of SNARE complexes and that SNAP29 and adaptor protein PLEKHM1 could be cleaved by viral 3C protease (Mohamud et al., 2018).

It has been shown that PV replication is associated with the formation of autophagosomes and independent of autophagic degradation (Richards and Jackson, 2012). Both viral proteins 2BC and 3A are required for the modification of LC3, which create double-membraned vesicles for viral replication (Taylor and Kirkegaard, 2007). Autophagy induction is promoted by two different upstream signaling pathways, AMP activated protein kinase (AMPK) activation, which leads to phosphorylation of ULK1, and mTOR inactivation, which causes the loss of the capability to phosphorylate ULK1 and disrupts the ULK1-AMPK interaction (Kim et al., 2011). From studies with a cell line of knockouts of autophagy-related genes, it’s determined that PV utilized ATG9 and LC3 for initiation of autophagy to promote viral intracellular growth (Abernathy et al., 2019).

It has been well-documented that the eukaryotic translation initiation factor 2 (EIF2S1) regulates protein synthesis by being phosphorylated under stress conditions (Kedersha et al., 1999). As a member of the activating transcription factor (ATF) subfamily, ATF4 is required for the transcriptional activation of a large number of autophagy genes in response to amino acid starvation and ER stress (B’chir et al., 2013). In the case of FMDV, viral protein VP2 was found to induce host autophagy via interacting with heat shock protein family B member 1 (HSPB1) to activate the EIF2S1-ATF4 pathway and inhibit the AKT-mTOR pathway, which could facilitate FMDV replication (Sun et al., 2018). In particular, expression of mutant VP2 protein reduced autophagy when this mutation affected viral infection and virulence. Besides, FMDV 2B protein can induce the host autophagy, which is associated with disrupting of Ca^2+^ content via viroporin activity (Ao et al., 2015).

Oxysterol binding protein (OSBP) regulates contracts of membranes containing phosphatidylinositol 4-phosphate (PI4P) (Mesmin et al., 2013). At the final stage of autophagy, PI4P is essential to the fusion of the autophagosome with lysosomes (Wang et al., 2015). Picornaviruses require specialized vesicular membranes (ROs) to replicate viral genomes by utilizing host OSBP/PI4P pathway. Several years ago, CVB3 was found to replicate on PI4P-riched organelles, and viral 3D polymerases could specifically bind to PI4P lipids (Hsu et al., 2010). The formation of RO membranes was later observed during RV, EV11, EV71, Achi virus (AiV), and EMCV infections (Spickler et al., 2013; Ishikawa-Sasaki et al., 2014; Roulin et al., 2014; Dorobantu et al., 2015; Strating and van Kuppeveld, 2017). Although the exact mechanisms how viruses utilized ROs for viral replication remains unclear, the formation of ROs requires viral 2BC and 3A protein to remodel intracellular membranes. Enterovirus 3A protein selectively manipulates the recruitment of PI4KB and GBF1, which provides a PI4P lipid-enriched membrane microenvironment to recruit 3D^pol^ from the cytosolic pool (Hsu et al., 2010). AiV requires PI4KB, Golgi protein acyl-coenzyme A binding domain containing 3 (ACBD3), and viral non-structural proteins 2B, 2BC, 2C, 3A, and 3AB forming a complex to enhance the generation of PI4P for viral replication (Ishikawa-Sasaki et al., 2014). Similarly, PV recruits PI4KB via 2BC and HRV 2B protein recruits PI4KB to replication membranes (Arita, 2014; Roulin et al., 2018). EMCV utilizes 3A protein to recruit PI4KA, another PI4K isoform, for promoting the generation of PI4P-enriched membranes. EMCV ROs depend on the activities of PI4KA and OSBP (Figure 3) (Dorobantu et al., 2015). HAV and ERAV replicate independently of both PI4KA and PI4KB. A detailed mechanism was reported in which the picornaviral 3CD protein was sufficient to induce PI4P synthesis, which indicates that this single protein has the capability to activate and commandeer the normal phospholipid biosynthetic pathway that is required for the viral life cycle (Banerjee et al., 2018). The autophagy-inducer increased PV infection, while the absence of essential genes for autophagy decreased viral infection (Jackson et al., 2005). The inhibitors of autophagosome maturation increased virion production of CVB3 (Wong et al., 2008). Thus far, growing evidence suggested that picornaviruses subverted the host autophagy pathway to promote replication. Identification of the mechanism used by picornaviruses for modulating the host membrane network and subverting autophagy will provide insights into novel mechanism of viral replication.

### Role of Autophagy in Viral Dissemination

Dismantling the cell membrane (cell lysis) has traditionally been considered a prerequisite way for the exit and transmission of picornaviruses. In recent years, however, increasing data have shown picornaviruses, including HAV, CVB3, PV, HRV and EMCV, that trigger autophagy and release in vesicles, referred to as non-lytic spread (Figure 3) (Feng et al., 2013; Bird et al., 2014; Robinson et al., 2014). Secretory autophagy is a non-degradative pathway in which autophagosomes fuse with the plasma membrane instead of lysosomes to release single-membrane vesicular structures filled with virions to the extracellular environment. The secretory autophagosomes and canonical degradative autophagosomes share conventional machinery; for example, the depletion of any autophagic genes including Atg12, LC3 and Beclin-1, results in decreasing viral release. In CVB3-infected cells, extracellular microvesicles (EMVs) containing infectious virions were released from infected cells (Robinson et al., 2014). A similar phenomenon, autophagosome-mediated exit without lysis (AWOF), was observed during poliovirus infection. Subsequent studies demonstrated via quantitative time-lapse microscopy that PV could spread to another cell without lysis, which was dependent upon components of the autophagy pathway (Bird et al., 2014). The non-enveloped viruses also harness non-lytic intercellular spread for pathogenesis These LC3-II positive autophagosomes containing virus did not fuse with the lysosomes and trafficked to the cell periphery where the outer membrane of DMVs fused with the plasma membrane. Moreover, it was found that the clustered PV particles were packaged into PS lipid-enriched vesicles and then transmitted from cell-to-cell via non-lytic release (Chen et al., 2015). This mode of transmission among cells could provide greater infection efficiency. Notably, the infection of vesicles containing PV particles is PS lipid- and PV receptor-dependent and not simply membrane-membrane fusion. However, a recent report showed that CV could trigger the release in an autophagosome-bound mitochondrion-virus complex, which targeted the DRP1-mediated fragmentation of mitochondria (a precursor to mitophagy). The suppression of the mitophagy pathway resulted in a marked decrease in virus production, suggesting that CVB subverts host mitophagy machinery to support viral transmission via releasing EMVs (Sin et al., 2017).

As an unusual member of the *Picornaviridea*, HAV has two mature infectious forms, enveloped (cloaked in host cell membranes) and non-enveloped. The enveloped viruses (eHAV), resembling exosomes to protect virions from antibody-mediated neutralization, are released from infected cells (Feng et al., 2013; Longatti, 2015). This process is dependent upon components of the cellular endosomal sorting complex required for transport (ESCRT). Interestingly, eHAV is the main form found in the blood of infected humans and animals; however, non-enveloped HAV is primarily found in the feces. This suggested that HAV utilized the host ESCRT machinery to escape from host immune responses and promote viral spread in the liver (Feng et al., 2013). A subsequent study revealed that eHAV, and not viral RNA exosomes or non-enveloped virus, was responsible for the activation of plasmacytoid dendritic cells (pDC) to produce type IFN- α at a limited level, suggesting that viral exosomes played a central role in immune evasion (Feng et al., 2015). Autophagy and other mechanisms of cytoplasmic transfer provide a link between viral egress and compromising host immune response.

## Conclusion

The researches on picornavirus and cellular factors have the potential to contribute strongly to increase our understanding of viral replication and pathogenesis. In this review, we described the current knowledge of the mechanisms of picornaviruses utilize to subvert the host apoptosis and autophagy for viral immune evasion, replication, and transmission by broad mechanisms. Several picornaviruses, such as PV, CVB3, HRV, and EMCV, can trigger the host apoptosis and subvert execution of apoptosis for their optimal replication via the cleavage of caspases and pro-apoptotic proteins, the disruption of Ca^2+^ homeostasis, and the suppression of nuclear-cytoplamic trafficking. Picornaviruses also share significantly conserved mechanisms of replication, for example, the requirement of remodeling the cellular membranes for viral replication. Depending on the source of cytoplasmic membranes, double-membrane autophagosomes following infection affect viral replication, and also serve as an essential player for the non-lytic release. Recently, more researches reveal the role of host pathways are subverted to build viral ROs. However, the exact relationship between autophagosomes and virus-induced membrane alteration is still being elucidated. Where picornavirus-induced ROs origin from? The contribution of apoptosis and autophagy to the different stages in the life cycle of picornavirus remains unclear. Currently, oncolytic virotherapy is regarded as a promising treatment approach, which exploited viral specific tissue tropisms to infect and kill tumor cells. The treatment of picornavirus has been developed to have greater tumor specificity in clinical trials. In this regard, the increased insights into picornavirus-host interactions can provide new opportunities to prevent viral infection and to develop oncolytic virotherapy.

## Author Contributions

DS conceived, designed, and wrote the manuscript. XW, MW, SM, AC, and XY revised the manuscript. RJ, SC, QY, YWa, DZ, ML, XZ, SZ, YWu, ZX, ZC, LZhu, QL, YL, YY, LZha, and XC helped with the manuscript. All authors read and approved the final manuscript for publication.

## Conflict of Interest Statement

The authors declare that the research was conducted in the absence of any commercial or financial relationships that could be construed as a potential conflict of interest.

## Abbreviations

ACBD3: acyl-coenzyme A binding domain containing 3
AIF: apoptosis-inducing factor
Aiv: Aichi virus
ATF4: activating transcription factor 4
AWOF: autophagosome-mediated exit without lysis
Bcl-2: B cell lymphoma 2
CV: coxsackievirus
dsRNA: double-stranded RNA
EMCV: encephalomyocarditis virus
EMV: extracellular microvesicle
ERAV: equine rhinitis A virus
EV1: echovirus 1
EV11: echovirus11
EV71: enterovirus 71
FADD: FAS-associated death domain
FIP2000: the focal adhesion kinase family-interacting protein of 200 kD
FMDV: foot-and-mouth disease virus
GSDMD: gasdermin D
HAV: hepatitis A virus
HSPB1: heat shock protein family B member 1
IFN: interferon
IPS: interferon promoter stimulator
IP3R: 1,4,5-triphosphate receptor
IRF: interferon regulatory factor
ISG: IFN-stimulated gene
MAVS: mitochondrial antiviral signaling proteins
MDA5: melanoma differentiation associated gene-5
MMP: mitochondrial membrane permeability
MOMP: mitochondrial outer membrane permeability
mTOR: mammalian target of rapamycin
NPC: nuclear pore complex
ORF: open reading frame
OSBP: oxysterol binding protein
PCD: programed cell death
PE: phosphatidylethanolamine
PI4P: phosphatidylinositol 4-phosphate
PI4KA: phosphatidylinositol 4-kinase III alpha
PI4KB: phosphatidylinositol 4-kinase III beta
PRR: pattern recognition receptor
PTB: polypyrimidine tract-binding protein
PtdIn3K: phosphatidylinositol 3-kinase
PS: phosphatidylserine
PV: poliovirus
RIG: retinoic acid inducible gene
RV: rhinovirus
SFPQ: splicing factor proline-and glutamine-rich
SG: stress granule
SNAP29: synaptosomal-associated protein 29
SNARE: soluble *N*-ethylmaleimide-sensitive factor attachment protein receptor
STX17: syntaxin 17
TGN: *trans*-Golgi network
TLR-3: Toll-like receptor 3
TNFR: tumor necrosis factor receptor
TOR: target of rapamycin
TRADD: TNFR-associated death domain
ULK1: uncoordinated 51-like autophagy activating kinase
UPS: ubiquitin-proteasome system
UTR: untranslated region
VDAC: voltage-dependent anion channel.
